# Supplementary material for: A Stretchable, Transparent, and Mechanically Robust Silver Nanowire–Polydimethylsiloxane Electrode for Electrochromic Devices
Source: Polymers (Basel). 2023 Jun 10;15(12):2640. doi: 10.3390/polym15122640 (PMC10305387; doi:10.3390/polym15122640)
Supplement: Supplementary file 1 [file polymers-15-02640-s001.zip › polymers-2383701-supplementary.pdf]

Supporting Information

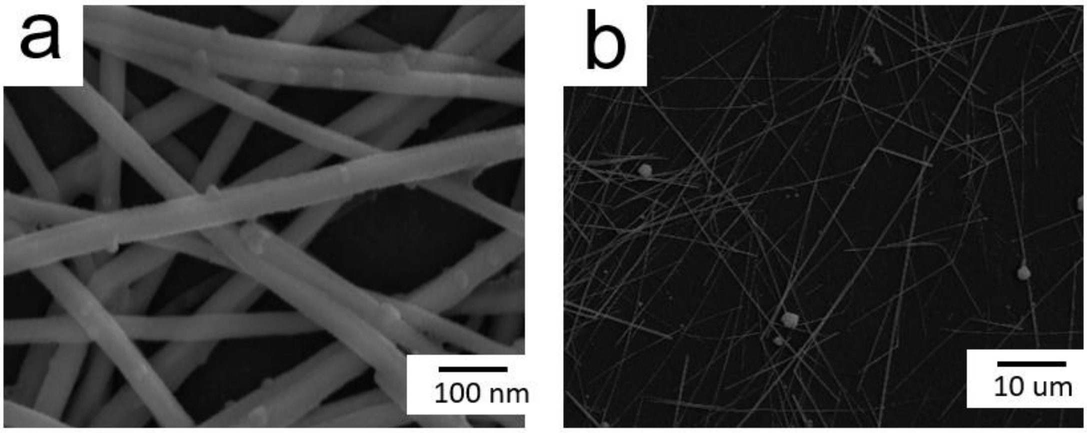

Figure S1 SEM images: AgNWs a and b.

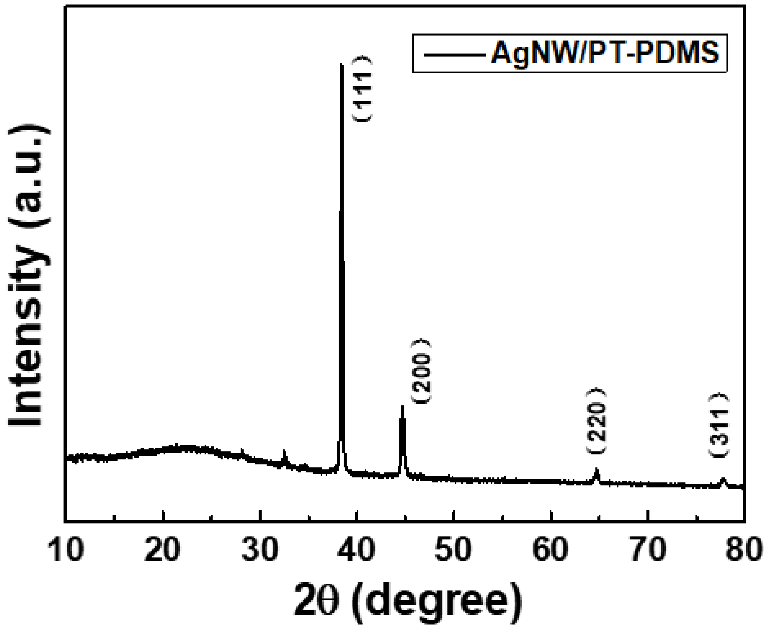

Figure S2 XRD patterns of AgNW/PT-PDMS.

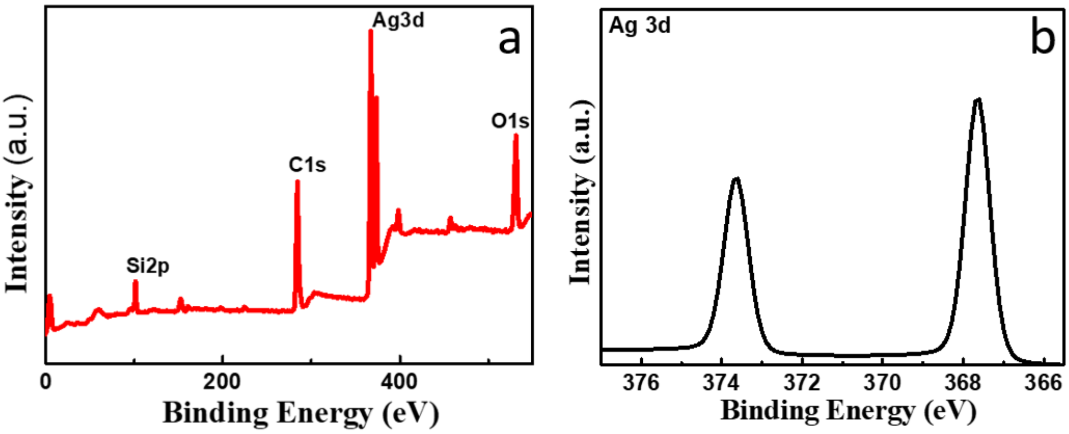

Figure S3 XPS spectra of AgNW/PT-PDMS (a) and Ag 3d.

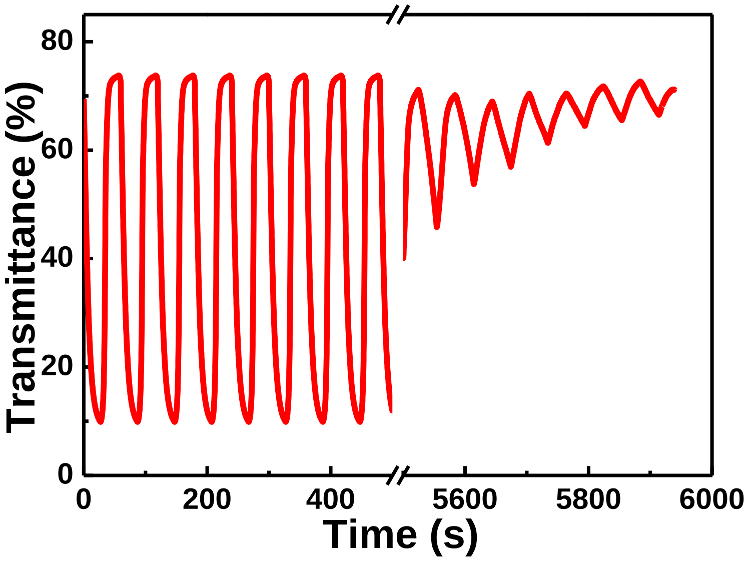

Figure S4 Electrochromic switching for 100 cycles of WO_3/AgNW/PT-PDMS electrodes.

Table S1 Optoelectronic properties and mechanical flexibility of Various Reported FTEs in comparison with AgNW/PT-PDMS

Samples	Transmittance (%)/Sheet resistance (Ω/sq)	Bending cycles	Peeling off cycles	Ref.
AgNW/PEDOT:PSS/PET	89.2/9.4	2000	30	[1]
MXene/AgNW-PVA	52.3/18.3	1000	-	[2]
AgNW-PVA	87.5/63	250	-	[3]
ZnO-PDMS	-	1000	-	[4]
AgNW/PDMS	75/20	1000	-	[5]
AgNW/PET	95/10	1000	-	[6]
AgNW/PDMS	-/0.5	1000	-	[7]
AgNW@TiO_2-PI	95/43.2	5000	100	[8]
AgNW/PT-PDMS	74/6	10000	500	Our work

Table S2 CHN analysis of AgNW/PT-PDMS

Samples	N(%)	C(%)	H(%)	S(%)
AgNW/PT-PDMS	0.00	35.76	5.96	0.00

References

(1) Li, X.; Yu, S.; Zhao, L.; Wu, M.; Dong, H. Hybrid PEDOT:PSS to obtain high-performance Ag NW-based flexible transparent electrodes for transparent heaters. *Journal of Materials Science: Materials in Electronics* **2020,** *31* (10), 8106-8115, DOI: 10.1007/s10854-020-03351-5.
(2) Zhou, B.; Su, M.; Yang, D.; Han, G.; Shen, C. Flexible MXene/Silver Nanowire-Based Transparent Conductive Film with Electromagnetic Interference Shielding and Electro-Photo-Thermal Performance. *ACS Applied Materials & Interfaces* **2020,** *XXXX* (XXX).
(3) Zeng, X. Y.; Zhang, Q. K.; Yu, R. M.; Lu, C. Z. A new transparent conductor: silver nanowire film buried at the surface of a transparent polymer. *Advanced Materials* **2010,** *22* (40), 4484-4488.
(4) Kumaresan, Y.; Min, G.; Dahiya, A. S.; Ejaz, A.; Shakthivel, D.; Dahiya, R. Kirigami and Mogul-Patterned Ultra-Stretchable High-Performance ZnO Nanowires-Based Photodetector. *Advanced Materials Technologies*.
(5) Liu, H. S.; Pan, B. C.; Liou, G. S. Highly transparent AgNW/PDMS stretchable electrodes for elastomeric electrochromic devices. *Nanoscale* **2017,** *9*.
(6) Highly Efficient and Bendable Organic Solar Cells with Solution-Processed Silver Nanowire Electrodes. *Advanced Functional Materials* **2014,** *23* (34), 4272-4272.
(7) Lin, Y.; Li, Q.; Ding, C.; Wang, J.; Yuan, W.; Liu, Z.; Su, W.; Cui, Z. High-resolution and large-size stretchable electrodes based on patterned silver nanowires composites. *Nano Research* **2022,** *15* (5), 4590-4598, DOI: 10.1007/s12274-022-4088-x.
(8) Huang, Y.; Tian, Y.; Hang, C.; Liu, Y.; Wang, S.; Qi, M.; Zhang, H.; Peng, Q. TiO2-Coated Core-Shell Ag Nanowire Networks for Robust and Washable Flexible Transparent Electrodes. *ACS Applied Nano Materials* **2019**.
